# The Iowa State Dental Society

**Published:** 1889-09

**Authors:** 


### PROCEEDINGS.

### The Iowa State Dental Society.

The twenty-seventh annual meeting of this body was held in Des Moines on May 7th, 8th, 9th, and 10th. About fifty members were present at the first session. The meeting was opened by prayer after which an address was delivered by Mr. Isaac Brandt, a prominent citizen of Des Moines in which he complimented the association very highly, and extended a very hearty welcome to the members to the city in which they had assembled. He spoke in very happy and complimentary terms of the profession. He gave expression to thoughts that indicated an unusual familiarity with dental matters, especially for a layman. He referred, with a high degree of intelligence, to the progress that has been made in dentistry during the past fifty years. Manifestly he is not a stranger to some of the arts of the dentist. At the close of these remarks a response was given by Dr. J. T. Abbott. He spoke of the great delight the members have experienced in finding Des Moines such a beautiful city and with so many attractions; he felt that the members would be more impressed with its attractions the longer they remained. At the close of these remarks the meeting adjourned till 2 P. M.

On the reassembling of the society the President, Dr. J. B. Monfort, of Fairfield, delivered the following address:

GENTLEMEN AND LADIES OF THE IOWA STATE DENTAL SOCIETY:—It is my pleasure to welcome you as we have assembled in this, the Capital City of our grand State, for our twenty-seventh annual meeting. The dental profession has not been at a stand-still during the past year. The minds of the men are so actively developing new theories, discovering new and easier methods of accomplishing certain ends, inventing instruments and appliances, that we almost become bewildered in our efforts to keep apace with the rank and file of our profession. Our journals bring us the thought and experiences of thinking men.

It is true that there is a great deal written that would have been as well had it been left unwritten. Theories advanced that are not well founded and instruments made that have little or no practical value. This, however, affords no excuse for our neglecting to use dental literature to gain information, and taking advantage of every real improvement. It requires an active mind to digest what we read and separate the true from the the false; good judgment and a thorough knowledge of the result to be accomplished is necessary to select instruments and appliances. We gain much knowledge by experience which is sometimes expensive, often extremely so; for proof of this we have only to search some dental office, where, hidden away in some secluded corner covered with dust, we find "a sure cure for pyorrhea alveolaris," or some other wonderful specific. Instruments without the marks of use, that were expected to fill a "long felt want," or appliances that were expected to revolutionize prosthetic dentistry. There is a tendency to burden ourselves with instruments. As the science of dentistry is progressing so rapidly are men apt to go to extremes, to take some one thing and make it their hobby, while other branches of as much importance are neglected. In so doing the profession is undoubtedly benefited by their experience but I fear their patients are the losers. We should be conservative in every thing, ready to adopt advanced ideas, but not until by such a course we feel confident that we shall serve the best interests of our patients. We have in common with those pursuing other vocations our offices invaded at frequent intervals by agents (commercial travelers) coming, as they no doubt believe, to do us good. Some of them are always welcome. The gentlemanly and accommodating agents representing our reputable dental depots, bringing us fresh supplies, instruments and appliances of the latest invention, are always welcome. But there are those who come (for example) with some device or preparation "warranted" to render the extraction of teeth painless, or a machine giving off lightning flashes that is said to accomplish the same result. Such things are offered to us by agents and by circulars through the mail. They take advantage of the selfish side of our nature, offering the "exclusive right" to use the same (by paying well for it of course) in the town or county in which we practice. They attempt to open our eyes upon a vision of our offices swarming with patients coming from far and near, our bank account increased one hundred per cent., while our brother across the corner is plodding along in the old way, his patients leaving him to struggle for bare existence. Such agents should be unwelcome to every professional man, not because their "stock in trade" is not what it is represented to be, or would not benefit us, but we should not encourage the granting of exclusive rights, for the reason that what is good for me and my patients, is good for my brother dentist and his patients. That men should be rewarded for their discoveries and inventions is just; but a preparation, method, or invention that is intended for the relief of suffering, or that is to improve the physical health of humanity should not be limited to a few, but put in reach of all. That there is no better means of assisting each other to keep abreast with the progress of dentistry than by meeting together as we now do, we are all agreed, as our presence testifies. The object of this society as expressed in our constitution is "to cultivate the science and art of dentistry; to promote among dentists mutual improvement, social intercourse, and good feeling, and to collectively represent and have cognizance of the common interests of the dental profession in our State." We do not realize, unless we look back over the past history of this society, what an educational institution it has been. Its influences can never be measured. It has been a constant stimulus to each individual member to press forward to a higher rank in his profession. It has been the means of establishing a dental law to regulate the practice of dentistry in our State, and a dental department in the State University. This being the fact, this society should regard with interest and no little degree of responsibility the success of these institutions. Our dental law is not what it ought to be, neither is it what the people should have. It was at the time of its passage perhaps the wisest law that could have been made, but it has served its time, a good work has been accomplished, the people are better cared for now than before the passage of the law. But the time has come when it should be changed so as to require a diploma from a reputable dental college in order to practice in the State. Temporary licenses should be a thing of the past; permitting students to practice after one course in college is but a step in advance of the six months office student being turned out as a full-fledged dentist. It not only reflects discredit upon the profession but it exposes the people to the mercies of the inexperienced student; they being ignorant of the fact until too late. When the question of changing the law was brought to the attention of one of our representatives his reply was, "The law should be changed, for the best dentists are poor enough."

That this society and all its interests may prosper, is the desire of all its members. By our association we shall still be led on to higher attainments. What is to be the future of our profession we do not know. Things that but a few years ago would have been pronounced as impossible to accomplish have been accomplished, proven practical, and the end is not yet. We regret that all practicing dentists of the State do not join hands with us in our efforts toward mutual improvement, for certainly this society does not require any thing of its members that should keep any professional man from reaping its benefits. And I trust that we will at this session have the pleasure of welcoming a large number into its membership.

Some discussion was had on the address of the President after which Dr. G. W. Miller, of Des Moines, read a paper on Pyorrhoea Alveolaris in which the position was taken that only about teeth having living pulps does this affection usually occur, and indeed that it rarely, if ever, takes place in connection with teeth after the pulp is destroyed. The paper was pretty fully discussed and the views presented were pretty severely criticized by all who spoke on the subject.

This subject being passed Dr. I. P. Wilson, of Burlington, read a very interesting paper on the Genesis of Oral Deformities. He discussed the various causes, considering at considerable length those of a pre-natal character and the premature extraction of the temporary teeth. So well was the subject presented that but little discussion was had on the paper.

Dr. E. Bergstreaser, of Cedar Rapids, read a paper on the Dental Pulp in which he took the ground that after completion of the tooth the pulp is no longer of service, and so whenever it becomes exposed, from whatever cause, it should in all cases be destroyed. This brought out some earnest discussion in which Drs Cochran, Ingersoll, Sudduth, Taft, and others engaged. In this discussion there was little or no concurrence with the views expressed in the paper.

The morning of the second day was occupied mainly in clinical work in which a number of very interesting operations were made. Dr. L. E. Rogers gave a practical test of the Bonwill mallet in filling teeth; Dr. Wm. Conrad gave his method of root-filling which elicited much interest; Dr. Angle gave an elaborate description and illustration of the uses and application of his appliances for correcting irregularities of the teeth in which he used maps, charts, and photographs, in the preparation and application of which great skill was shown. Dr. Angle certainly stands abreast with the foremost in the practical part of this work.

Dr. C. Thomas demonstrated an excellent method of porcelain filling.

Dr. W. H. Baird gave a demonstration in inserting porcelain crowns. Dr. C. S. Searls took bridge-work for his clinic, and made a very interesting and instructive demonstration. Dr. T. E. Weeks showed the method of preparing cavities in teeth for filling, without pain, in which he was in a marked degree successful. Dr. C. J. Peterson showed the proper method of using Perry's Separator.

At the afternoon session Dr. T. E. Weeks, being called upon, gave a very interesting talk upon the treatment and formation of cavities in the proximal surfaces of the six anterior teeth, using a large model to illustrate his remarks. Following this each one who had operated in the forenoon was called upon to make any additional description of the operation performed by him, and in answer to this almost every one made some explanatory remarks, descriptive of what had been done. Questions were asked and answered; explanations given, and the work of the morning was pretty well gone over. This discussion having been concluded Dr. Wm. Conrad, of St. Louis, asked for the floor that he might present the claims of the American Dental Protective Association. In his remarks he asked for it the hearty cooperation and support of every dentist who has the interest and welfare of his chosen profession at heart. It proposes to protect all its members from unjust demands and prosecutions by the International Tooth-Crown and Bridge Co., and indeed, from any and all organizations who may be disposed to press unjust demands and exactions upon the members of the profession. An emergency is now upon the profession through the demands of the International Tooth-Crown Co., based upon patents, the legality of which is called in question, or at least the validity of which has not been fully established. This company is collecting large sums of money of those who yield to its demands, and are using it to bring to their terms the entire profession. Will you submit to this kind of procedure? I trust that every one here will become a member of the Dental Protective Association, and receive the protection which it can afford.

After this some discussion was had upon the management of the Dental Department of the University of Iowa. This, however, was of special interest only to the profession in that State and need not be given here.

The evening session was occupied by Dr. W. X. Sudduth, of Philadelphia, on lesions of the dental pulp. The lecture was well illustrated by a fine series of micro-photographs projected on the screen with the stereoptican; it is not possible to do justice to either the lecture or the illustrations here, only those who hear and see can have any adequate appreciation or idea of the subject as presented, and those who absent themselves from our association can have no conception of the loss they sustain, nor how far they are sure to be left in the rear.

On the morning of the third day the work was taken up according to the programme. Prof. Brophy removed a tumor from the mouth, which was a matter of much interest to all who witnessed it. After this a paper was read on "Prothetic Dental Art," by Prof. A. O. Hunt, this was a very complete and elaborate presentation of the subject; one that has cost years of study and investigation. He began with the study of the human face; this he declared to be a complicated and difficult one; it has many phases. Among other things the physiognomist directs his attention to those features that indicate the hidden characteristics; it is important that dentists should be artists as well as good technical manipulators. The natural features should be maintained, or if lost, restored as perfectly as possible. There are rules of harmony that should be observed and cultivated by every one who undertakes to manipulate the human features, or engages to restore lost parts. Failure in this particular indicates that one has missed his calling and should bring himself up to a satisfactory attainment in this respect, or turn his attention to some more congenial pursuit. Many ignore or neglect the highest possibilities in sight and do not show that they have ever had a thought above the commonplace and sordid one. The same care and thought ought to be given to dental work that is bestowed upon fine art and mechanism.

If the Apollo Belvedere should present himself with teeth gone, how many dentists could succeed in complete restoration and the securing a perfect effect? In many persons we find the clear typical human face, while in many others are found more or less the marked animal features. Following this was a scholarly and artistic description of leading facial outlines and characteristics, and the necessity of a thorough study of these was dwelt upon with strong emphasis. The paper was illustrated fully with plaster casts, photographs, etc.

The paper was discussed by Dr. Brady, of Minneapolis, and Dr. Weeks of the same city, both of whom spoke in very high terms of the paper and the presentation of the subject, and most significantly emphasized its importance. The fourth and last day was devoted mainly to clinics. Dr. E. L. Brooks, of Vinton, filled the proximate cavity of a molar, using the Herbst method of rotary motion and hand pressure.

Dr. E. T. Weeks demonstrated the method of removing a pulp from a tooth by the use of electricity.

Dr. W. P. Dickinson filled a very difficult compound cavity with iridium gold, using the hand mallet.

Dr. Angle made very clear his method of correcting irregularity, fully explaining his appliances and their use. The final paper of the meetings was read by Dr. G. W. Clark, of Cedar Rapids, on Dental Education, in which he urged the importance, and indeed the necessity, of educating the people to the importance of appreciating good dental work. A work so important for the welfare of humanity should be well understood and appreciated by those who require such service. The amount of suffering and discomfort relieved and prevented by dentistry is incalculable, and the people should know what the dental art, guided by science, is capable of doing for them. He urged with special force the thorough education of the dentist before he is allowed to practice, and after this his constant effort is required to keep up with the times.

The paper was discussed by Dr. M. G. Jennison, of Minneapolis, who emphasize most strongly the points made in the essay. One of the best and most appropriate things done by the meeting was the election to honorary membership of Mrs. W. P. Dickinson, of Dubuque, Iowa. Mrs. D. has a far better knowledge and appreciation of what dental science and art is, than thousands who are attempting to practice; indeed she is well up, and intensely interested, in every thing relating to the profession.

It is but just to say that the twenty-ninth meeting of the Iowa Dental Society was a successful and profitable one, and is a grand prophesy for the future.

---

Mouth Wash.—The following wash for shrinking of the gums is given by various French journals of Pharmacy: Tannic acid, 8 gm.; tr. iodine, 5 gm.; iodide potass., 1 gm.; tr. myrrh, 5 gm.; rose-water, 200 gm.; mix. A teaspoonful in a third of a tumbler of water.—*Canada Lancet.*

---

After the Vacation.—Coles—"Back from the country?"

Boles—"Yes."

Coles—"Feel recruited?"

Boles—"Haven't been back long enough to feel the benefits yet."—*Life.*

